# Systematic review of brucellosis in Kenya: disease frequency in humans and animals and risk factors for human infection

**DOI:** 10.1186/s12889-016-3532-9

**Published:** 2016-08-22

**Authors:** J. Njeru, G. Wareth, F. Melzer, K. Henning, M. W. Pletz, R. Heller, H. Neubauer

**Affiliations:** 1 Institute of Bacterial Infections and Zoonoses, Friedrich-Loeffler-Institut (FLI), Naumburger str. 96a, 07743 Jena, Germany; 2 Center for Infectious Diseases and Infection Control, Jena University Hospital, 07740 Jena, Germany; 3 Centre for Microbiology Research (CMR), Kenya Medical Research Institute, P. O. Box 19464-00202, Nairobi, Kenya; 4 Institute for Molecular Cell Biology, Center for Molecular Biomedicine, Jena University Hospital, 07745 Jena, Germany; 5 Faculty of Veterinary Medicine, Benha University, Moshtohor, Egypt

**Keywords:** *B. melitensis*, *B. abortus*, Seroprevalence, Epidemiology, Kenya

## Abstract

**Background:**

Brucellosis is a debilitating zoonotic disease affecting humans and animals. A comprehensive, evidence-based assessment of literature and officially available data on animal and human brucellosis for Kenya are missing. The aim of the current review is to provide frequency estimates of brucellosis in humans, animals and risk factors for human infection, and help to understand the current situation in Kenya.

**Methods:**

A total of accessible 36 national and international publications on brucellosis from 1916 to 2016 were reviewed to estimate the frequency of brucellosis in humans and animals, and strength of associations between potential risk factors and seropositivity in humans in Kenya.

**Results:**

The conducted studies revealed only few and fragmented evidence of the disease spatial and temporal distribution in an epidemiological context. Bacteriological evidence revealed the presence of *Brucella (B.) abortus* and *B. melitensis* in cattle and human patients, whilst *B. suis* was isolated from wild rodents only. Similar evidence for *Brucella* spp infection in small ruminants and other animal species is unavailable. The early and most recent serological studies revealed that animal brucellosis is widespread in all animal production systems. The animal infection pressure in these systems has remained strong due to mixing of large numbers of animals from different geographical regions, movement of livestock in search of pasture, communal sharing of grazing land, and the concentration of animals around water points. Human cases are more likely seen in groups occupationally or domestically exposed to livestock or practicing risky social-cultural activities such as consumption of raw blood and dairy products, and slaughtering of animals within the homesteads. Many brucellosis patients are misdiagnosed and probably mistreated due to lack of reliable laboratory diagnostic support resulting to adverse health outcomes of the patients and routine disease underreporting. We found no studies of disease incidence estimates or disease control efforts.

**Conclusion:**

The risk for re-emergence and transmission of brucellosis is evident as a result of the co-existence of animal husbandry activities and social-cultural activities that promote brucellosis transmission. Well-designed countrywide, evidence-based, and multidisciplinary studies of brucellosis at the human/livestock/wildlife interface are needed. These could help to generate reliable frequency and potential impact estimates, to identify *Brucella* reservoirs, and to propose control strategies of proven efficacy.

## Background

Brucellosis is one of the most common zoonoses worldwide. The disease has been eliminated substantially in several developed countries including Australia, Japan, New Zealand, Canada, and some European countries, but it remains a major public health problem in Mediterranean region, Middle East, Africa, Latin America, and parts of Asia [1]. The disease affects are wide range of domestic and wild animals causing abortions, reduced milk yield, and infertility resulting in tremendous economic losses in livestock production [2]. In humans, *Brucella* cause systemic infections with an acute, subacute, or chronic relapsing course. Clinical presentation of human brucellosis is nonspecific and highly variable. Patients commonly have a wide range of symptoms including undulant fever, headache, chills, myalgia, and arthralgia. The disease is also associated with abortion, orchitis, acute renal failure, endocarditis, splenic abscess, spondylitis, arthritis, and encephalitis [3–5]. Up to date, the genus *Brucella* includes 12 accepted nomo-species but only *B. melitensis, B. abortus, B. suis*, and in rare cases *B. canis* are considered to be human pathogens. The zoonotic potential of the remaining species has not yet been confirmed [6, 7]. Animals and their products are the main source of human brucellosis. Transmission occurs via the consumption of unpasteurized dairy products or direct contact with infected animals through skin abrasions or mucous membranes [8]. Individuals with occupational livestock contact such as farmers, veterinarians, abattoir workers, and livestock keepers are at high risk of infection. The families of these groups are also at high risk as domestic exposure may be unavoidable when animals are kept in close proximity to living areas [1, 8, 9].

Diagnosis of brucellosis in sub-Saharan Africa is often challenging to clinicians due to the wide spectrum of clinical manifestations and lack of reliable diagnostic tests. This frequently results in misdiagnosis as malaria or other febrile diseases. Thus brucellosis remains severely underreported [10–12]. Approximately, about 500,000 new cases are estimated to occur every year globally [1]. Despite this and the high burden of the disease in many low-income countries, the disease does not attract the appropriate attention of health systems. Thus brucellosis is presently classified as one of the top neglected zoonosis by World Health Organization (WHO) [13].

In sub-Saharan Africa, brucellosis is endemic in countries with extensive pastoral production systems where surveillance and control are rarely implemented. It is often ignored in humans potentially leading to considerable suffering of the patients [12]. In Kenya, livestock production is a rapidly growing economic activity for communities that live in the high rainfall areas for intensive dairy production. Agro-based pastoralism, extensive pastoralism, and commercial beef production are common in the arid and semi-arid lands (ASAL) [14]. However, the high incidence of tropical vector borne diseases and re-emerging infectious diseases in animals hinders animal production and international livestock market due to trade sanctions [15].

Though the first case of brucellosis was described in Kenya in 1916, scanty data are available on the disease burden estimates and a comprehensive, evidence based assessment of the literature is missing. A better understanding of the epidemiology of brucellosis (prevalence estimates, affected host species, risk factors, potential reservoirs, and prevalent *Brucella* species) would be important for recommending a prevention and control strategy.

In this systematic review, we evaluate and summarize relevant articles reporting on the presence, frequency, and control of brucellosis in humans and animals in Kenya. We also present findings on the associations between potential risk factors and seropositivity in humans to verify the current situation of brucellosis in the country.

## Methods

### Systematic review protocol

**Fig. 1:**
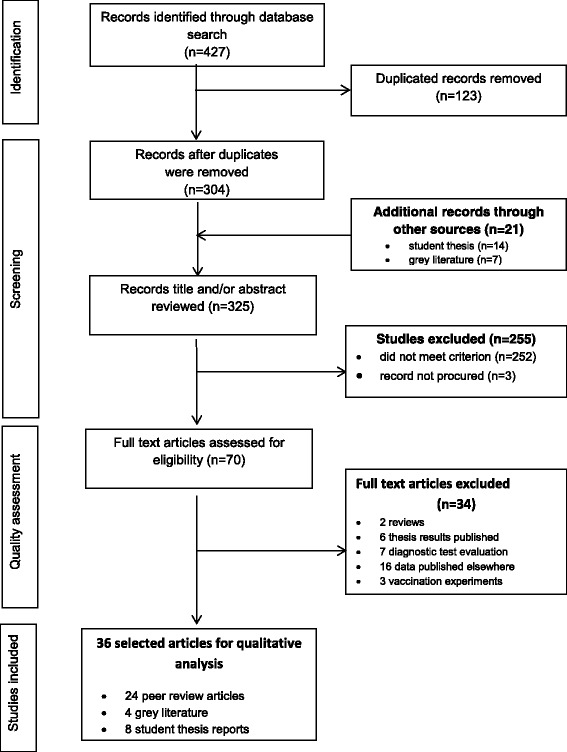
Search strategy and paper selection flowchart showing the numbers of articles at each stage of the systematic review

A systematic review was conducted using a predefined protocol based on PRISMA [16] guidelines including: (i) literature search to identify potential articles of relevance, (ii) assessing the relevance of the articles, (iii) quality assessment and (iv) data extraction. Figure 1 summarizes the number of articles that fulfilled the necessary criteria at each step.

### Literature search and data collection

Search in the database search engines (PubMed, Google scholar, African Journals Online, Science Direct, Cabdirect) was undertaken using different search terms and Boolean Operators including,

(i) Brucellosis OR *Brucella* OR Brucel* OR Zoonoses OR Zoonotic diseases OR zoonos*
  AND
(ii)  Kenya OR Africa.
  AND
(iii)  Humans OR Wildlife OR Domestic AND Ruminant OR (cattle OR bovine) OR (Camel OR Dromedary) OR (Sheep OR ovine) OR (Goat OR Caprine).
  AND
(iv)  Prevalence OR Incidence OR prevention AND control OR Risk Factors.

We also applied wildcard symbol (*) in some of the searches. During the searches, the combinations were either relaxed or broadened to capture more articles or were restricted to refine the number of resulting articles. Other related articles emerging during the searches were also considered as sources of additional information. Bibliographies of selected papers were also reviewed.

Similar search terms were used for obtaining additional information from the published grey literature materials. Publications not available on-line were searched through personal visits to the Kenyan university libraries and government departments. No time limits were set. The studies or reports unrelated to the predetermined criteria were then excluded. These included those describing studies conducted outside Kenya, immunology experiments, incorrect pathogens, duplicate data published elsewhere, reviews or lay media content and textbooks/manuals.

We used a broad inclusion criterion to allow us to identify the presence of the disease, limitations of the diagnostic tests used, potential risk factors, *Brucella* host ranges and reservoirs, transmission dynamics, disease diagnostics, control programmes, and the gaps from which lessons can be drawn.

### Data screening

The abstracts of the retrieved articles were screened by the primary author based on the following criteria:

- The article reported original data on brucellosis in Kenya,
- The article provided information on occurrence ([sero-prevalence] and/or incidence) or outbreak report of *Brucella* spp. in animals and/or humans,
- The article described case reports/series of *Brucella* spp. infection in animals and/or humans,
- The article reported on control programmes,
- The publication investigated the associations of potential predisposing risk factors and *Brucella* spp. infection or seropositivity in humans.

For articles whose relevance could not be determined by reading the abstract alone, full texts were retrieved and the quality assessment of the article conducted.

### Quality assessment and data extraction

A full text analysis for each publication was done by two independent reviewers using a pre-designed data extraction form and the quality assessment for eligibility was conducted for each article independently.

Each of the reviewers assessed the ability of the article to fulfill the following criteria:

(i) A description of the study design or the sampling strategy and approach applied,
(ii)  For prevalence studies, case studies or case series, at least one classical diagnostic test was applied,
(iii)  The study population and sample size was described for epidemiological studies,
(iv)  For epidemiological studies, the study region and period were specified,
(v) For studies investigating the risk factor for *Brucella* infection, estimates of the strength of association are provided,
(vi)  Possibility of the reviewer to obtain information on animal production and management system.

Those that fulfilled the quality assessment were considered to be of sufficient quality to provide evidence of the occurrence of brucellosis in different host populations in Kenya or possible predisposing risk factors. Publications describing brucellosis investigations were included even if statistical analyses applied were not sound to promote data acquisition.

Data was then extracted on:

- Animal species involved,
- *Brucella* species or their biovars identified,
- Relative risk and odds ratio estimates of the strength of association between *Brucella* seropositivity in humans and potential risk factors,
- Disease prevention or control methods used,
- Type of study,
- Study outcome and reviewer comments,
- Location of study,
- Study population,
- Study period,
- Sampling approach (probability or nonprobability sampling),
- Diagnostic test used,
- Sampling strategy,
- Bias and/or gaps in sampling method described.
- The sampling strategy for animals was categorized into herd, flock, individual, abattoir, meat market, and milk markets. For the farm studies, the livestock production system was identified and where multiple surveys were reported in a single study, each survey was listed separately.

## Results

### Data acquisition

The initial database searches revealed 427 research articles and after removing duplicates and those that did not meet the eligibility criterion, 36 articles remained for data extraction and qualitative analysis. These included thirteen articles reporting the occurrence of brucellosis or providing estimates of brucellosis frequency in humans [17–29], three studies on livestock and humans [30–32], and two studies reported on potential risk factors for human brucellosis seropositivity [33, 34]. Fifteen studies reported brucellosis in livestock [35–49], two in wildlife [50, 51], and one article in mixed livestock and wildlife herds [52] Fig. 1.

The range of the years of publication was 1931–2016 with a median of 2008 and 1990 for human and animal studies, respectively. The studies were heterogeneous in terms of study design within human groups or animal subpopulations under investigations and sample size. The majority of the studies did not report probability sampling and others did not specify the sampling strategy used at all. Thus data were only extracted, summarized, and organized in a qualitative manner.

To summarize and compare data of *Brucella-*specific antibody based studies, one test result value (that met the recommended titer cut-off of the respective test method used) per study is reported in the following preferential order: Rose Bengal Plate test (RBT), Enzyme-Linked Immunosorbent Assay (ELISA), Complement Fixation Test (CFT), Serum Agglutination Test (SAT). The rationale for this preferential selection is based on the proposed sensitivity/specificity superiority generated through meta-analytical logistic regression model algorithms that allows adjustment for confounding factors for specificity and sensitivity of the classical *Brucella* diagnostic tests [53]. For studies where milk was screened with Milk Ring Test (MRT), these values are reported.

Among livestock studies reporting disease frequency estimates, we present only prevalence ranges after taking into consideration the heterogeneous nature of the retrieved studies.

**Fig. 2:**
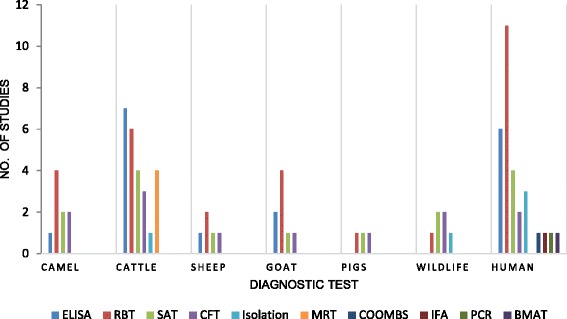
The number of studies in which specific diagnostic tests were used. The data table corresponds to the total number of studies that have employed each test for each species. The overall number of studies is greater than the total number of papers reviewed because many studies applied more than one test method. ELISA: Enzyme-Linked Immunosorbent Assay, RBT: Rose Bengal Plate Test, SAT: Serum Agglutination Test, CFT: Complement Fixation Test, IFA: Immuno Fluorescence Antibody Assay, MRT: Milk Ring Test, BMAT: Brucella Micro-Agglutination Test

Most studies screened blood or milk with multiple serological tests and reported disease frequencies based on the results of each individual test. One and five studies, respectively, reported brucellosis based on molecular and bacteriological evidences. A summary of the studies which used classical serological tests, bacterial isolation and molecular methods is shown in Fig. 2.

### Evolution and spatial distribution of publications

**Fig. 3:**
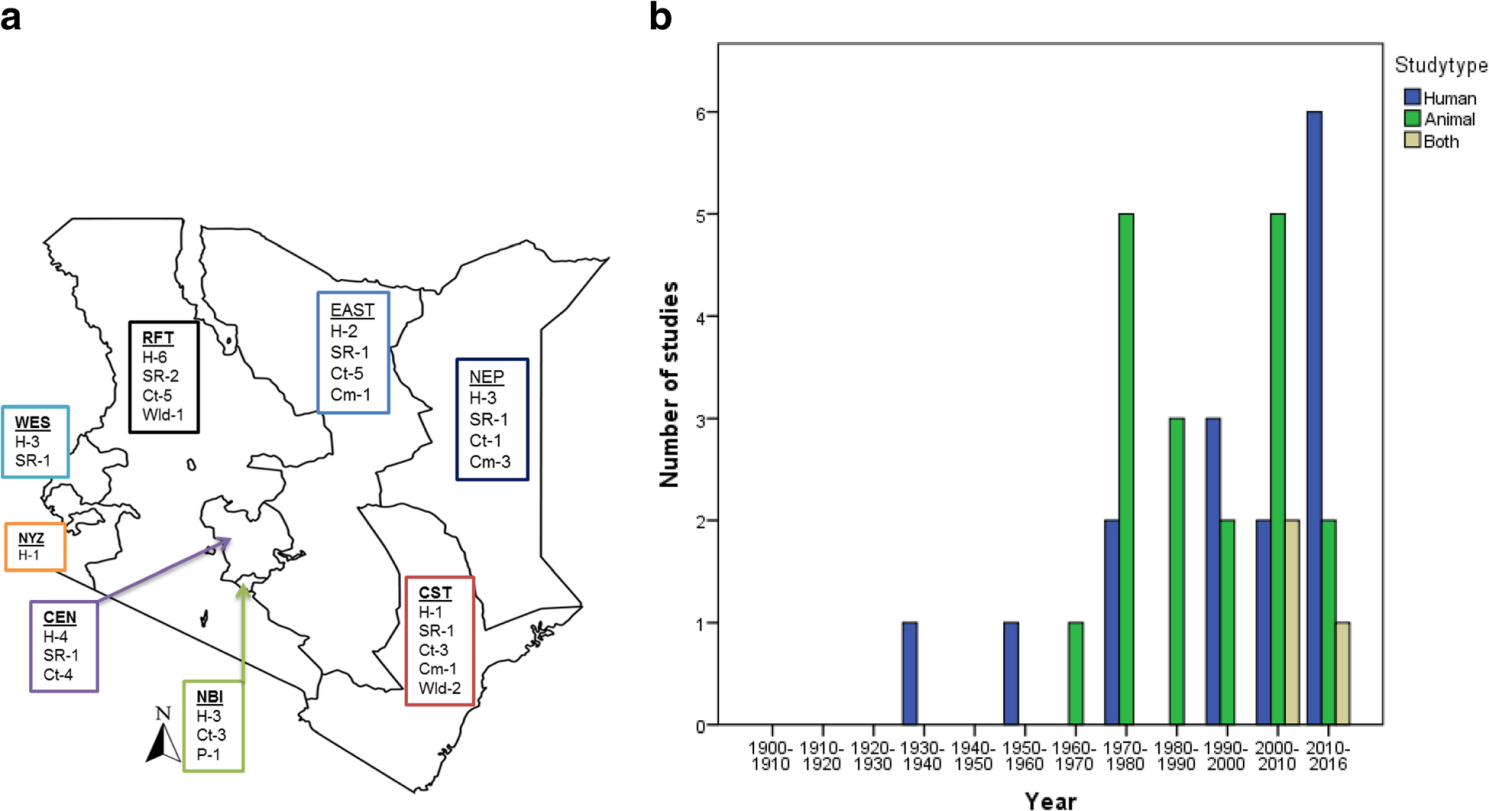
**a** Map of Kenya showing the distribution and number of brucellosis studies conducted in the last 100 years according to host species per province. Kenyan Provinces: CEN (Central), CST (Coast), (EAST) Eastern, NBI (Nairobi), NEP (North Eastern), WES (Western), NYZ (Nyanza), (RFT) Rift Valley. Host species: H: human, SR: small ruminant, Ct: cattle, Cm: camel, P: pig, Wld: wildlife. **b** The number of studies conducted per decade in Kenya according to hosts, included in the systematic review

Brucellosis was first reported in Kenya in 1916 but the first laboratory confirmed case was reported in 1931. The period from 1970 to 2010 was characterized by a progressive increase in the number of animal and human studies. Between the years 2010 and 2015, a sharp increase in the number of human studies occurs while the number of animal studies decreased. All studies were regional except one that conducted a national seroprevalence survey of human brucellosis in 2007. The distribution of studies by animal species and humans in each of the Kenyan provinces is shown in Fig. 3a while the number of studies according to year of publication and host investigated is shown in Fig. 3b.

### Human brucellosis

The frequency of brucellosis in humans was investigated by 16 studies including 10 hospital-based studies, 2 population based studies, 3 studies among high risk occupational groups, and 1 outbreak investigation. Of these, only one study [24] described a nationwide population-based surveillance for human brucellosis. The study found an estimated national seroprevalence of 3.0 %. No case was found in Nairobi and Nyanza provinces, while low levels of exposure were found in other provinces. Relatively higher seroprevalence was found in the Northeastern province. Of the other studies, evidence of human brucellosis was reported in six of the eight Kenyan provinces. Three early case series/reports described brucellosis in patients treated and followed up at Nairobi and Machakos hospitals. The disease was found in Africans, Asians, and Europeans [19, 25, 29]. A study by Paul et al. (1995) reported brucellosis in HIV and non-HIV infected patients of a study cohort in Nairobi [27]. Ari et al. (2011) reported a brucellosis associated outbreak of acute febrile illness among pastoralists in a remote part in Northeastern Kenya [17]. Other sero-epidemiological studies revealed high prevalence of brucellosis in humans from five counties: Kanjiado (Rift valley province), Narok (Rift valley province), Marsabit (Eastern province), Turkana (Rift valley province), Machakos (Eastern province), and Garissa (Northeastern province). Comparatively, low seroprevalences were found in Busia (Western province), Nairobi (Nairobi province), Kiambu (Central province), and Naivasha (Rift valley province).

The animal-human linked study by Osoro et al. (2015) found evidence of strong association between human and animal seropositivity [32]. Table 1 shows the summary of brucellosis studies on humans and Fig. 3a, the number of studies conducted per province.

**Table 1:** Summary of studies investigating the occurrence of brucellosis in humans in Kenya deemed as relevant to be included in this systematic review

Population	County	Diagnostic test	Complementary tests	Study outcome		Ref
				Diseases frequency^a^	Risk factor	
Apparently healthy	National	ELISA	NA	**3 (1.0–5.0)**	Yes	[24]
	Nairobi Central Coast Eastern N/eastern Nyanza Rift Valley Western		NA	**0** **1.1 (0.0–2.7)** **1.0 (0.0–2.9)** **1.5 (0.0–4.3)** **10.3 (0.0–21.8)** **0** **2.8 (0.0–7.0)** **0.5 (0.0–1.6)**		
Apparently healthy	Kiambu Kajiado Marsabit	ELISA	NA	**2.4 (1.9–30)** **15.3 (10.5–21.8)** **46.5 (39.0–54.1)**	Yes	[32]
Outbreak patients	Garissa	RBT/BMAT	ELISA,CFT	5 of 12 outbreak cases	No	[17]
Hospital patients	Narok	RBT	NA	21.2 (13.8–35.9)	No	[23]
Hospital patients	Machakos	RBT	SAT	39 patients case series report	No	[25]
Hospital patients	Machakos	RBT	CFT,SAT	**10.4** ^**b**^	No	[26]
Hospital patients	Garissa	RBT	PCR	**31.8** ^**b**^	Yes	[21]
High risk pastoralist population	Isiolo	QS	NA	QS	Yes	[33]
Hospital patients	Nairobi	RBT	ELISA	**5 (1.4–9.4)**	No	[22]
Hospital patients	Nairobi	ELISA	Isolation	2 BM isolates and 21 seropositive from study cohort	No	[27]
High risk pastoralist population	Turkana	RBT	ELISA	**17 (13.1–22.4)**	Yes	[30]
Hospital patients	NS	RBT	Isolation	70 isolates (64BM,6BA) from case reports	No	[29]
Hospital patients	NS	Isolation	NA	1 BA isolate from a case report	No	[19]
Hospital patients	Kiambu Narok	RBT/SAT	IFA	**3.2 (1.1–4.5)** **14.4 (8.0–18.9)**	No	[31]
High risk occupational groups	Nairobi Naivasha	SAT	NA	**2 (0.5–4.5)** **7 (4.1–14.6)**	No	[20]
High risk pastoralist population	Marsabit	QS	NA	QS	Yes	[34]
High risk occupational groups	Busia	RBT	NA	**0.1 (0.007–0.8)**	No	[18]
Hospital patients	Busia	RBT	BPAT, Coombs, SAT	**0.6 (0.04–0.9)**	NO	[28]

^a^Seroprevalence estimates (bold) with the corresponding 95 % Confidence Intervals or outcome from outbreak investigation/case reports/case series

^b^95 % Confidence Interval not provided, *NA* not available, *Ref* reference, *BA B. abortus*, *BM B. melitensis, ELISA* enzyme-linked immunosorbent assay, *RBT* rose bengal plate test, *SAT* serum agglutination test, *CFT* complement fixation test, *IFA* immuno fluorescence antibody assay, *BMAT Brucella* micro-agglutination test, *NS* not specified, *QS* qualitative studies without laboratory investigations (Cases defined as individuals diagnosed and treated for brucellosis in the past one year)

RBT was the primary diagnostic test applied in 11 (68.8 %) studies. Of these, seven studies had at least a complementary serological test including: CFT, SAT, ELISA, IFA or Coombs test. One study demonstrated evidence of *Brucella* infection in febrile patients using PCR, while three case report or series provided bacteriological evidence of *Brucella* infection in human patients Table 1.

### Animal brucellosis

A total of 21 studies investigated the frequency of brucellosis in different animal species from various production systems (Table 2). In camels, the seroprevalence ranged from 4.6 to 38 % from pastoral managed camel herds in northern Kenya, and 8.0 % in a single extensively managed commercial ranch in the coast region [32, 39, 46, 48, 52].

**Table 2:** Summary of brucellosis studies in animals in Kenya identified as relevant to be included in this systematic review

Population production system	Test (No. of studies)^a^							% Range of seroprevalence^b^							Reference
	Camel	Cattle	Sheep	Goat	Milk	Pigs	Wildlife	Camel	Cattle	Sheep	Goat	Milk	Pig	Wild life	
Pastoral	E(1), R(3),S(1), C(1)	E(3), R(3),S(3), C(2)	E(1)	E(2) R(1)			S(1), C(1)	10.3–38.0	9.9–16.9	11.9	13–16.1			18–30	[30, 32, 37–39, 43 45, 46, 48, 51]
Agro-pastoralist	-	E(1), R(1), S(1)	E(1), R(1)	E(2), R(1)	E(1), M(1)			-	3.3-10.0	0–3.4	3.6–5.0				[32, 36]
Small holder	-	E(2), S(1)	E(1)	E(1), R(1)	M(3) E(2)		-	-	0.8-9.0	2.4	0–1.3	0–13.6			[32, 35–37, 40, 42 44, 45]
Abattoir						R(1) S(1), C(1)							0.2		[47]
Extensive	R(1), S(1) C(1)	R(2), S(1), C(1)	R(1) S(1) C(1)	R(1) S(1), C(1)			R(1), S(1), C(1)	8	17& 7/10 cases	0	0	-	-	0-14	[49, 52]
Not specified		I(1)			M(1) E(1)		I(1)					0–10			[41, 50]

^a^Range of diagnostic tests and respective number of studies for each test on which individual prevalence values have been based

^b^Proportions of animals positive for brucellosis based on the prioritized tests selection criteria

*E* enzyme-linked immunosorbent assay, *R* rose bengal plate test, *s* serum agglutination test, *C* complement fixation test, *F* immuno fluorescence antibody assay, *M* ring test, *I* bacterial isolation

In cattle, the seroprevalence from pastoral and agro-pastoral managed herds ranged from 9.9 to 15 % in the studied regions of Northeastern province [38], Kanjiado (south Rift valley) [43], Turkana (north Rift valley) [30], and Marsabit and Samburu (upper Eastern) [32, 37, 45]. The seroprevalence was relatively higher (10.7 %–14.9 %) in Samburu, while that from Kilifi increased from 4.1 to 9.0 % in two studies conducted in the same regions about 20 years apart [37, 45].

Studies of cattle from small holder production system regions found seroprevalences below 2.5 % in Kiambu [32, 37, 45]. High seroprevalence of brucellosis and a probable brucellosis associated outbreak were revealed in cattle from extensive beef production commercial ranches in the Coast and upper Eastern regions, respectively. The cattle shared the grazing pastures with domesticated wild animals [49, 52].

In goats, the high seropositivity was found from pastoral managed herds in north Rift Valley and the upper Eastern regions [30, 32]. Low seroprevalence of brucellosis was found in goats and sheep from small holder farms in Kiambu (Central). No case was detected in a single seroprevalence study conducted in Busia (Western) Kenya [35]. Similarly, low seroprevalences were reported in small ruminants from agro-based pastoralists’ herds in Kanjiado while none was found in an extensive mixed herd ranch in the coastal region [30, 32].

Serological evidence of *Brucella* infection was found in 0.2 % pigs in an abattoir study in the Central province [47], whilst an early study found high seroprevalence in wildebeest and African buffalo from Maasai Mara (south Rift Valley) [51]. The animals shared grazing and watering areas with cattle of Maasai communities who are predominantly pastoralists.

In milk based studies utilizing ELISA and MRT assays, low brucellosis detection was reported in raw milk samples sold in small units in urban markets in Nairobi and Eldoret (central Rift Valley). In contrast, more positives were reported from milk originating from the extensive production units in Nakuru (south Rift Valley) [36, 40, 42]. Nyaga, (2010) found high seroprevalence of *Brucella* antibodies in milk obtained from informal market agents in Narok and Kiambu in Central province [31]. In Nairobi, low bovine brucellosis prevalence was reported in milk samples from non-dairy farming households and from dairy farming households [40].

RBT was used in 10 of 19 studies and was either complemented with ELISA or SAT and CFT in one and seven studies, respectively. Consumption of locally fermented milk products prepared from raw milk was commonly reported among rural and urban households across Kenyan regions, whereas consumption of raw milk was only reported among rural households [36, 40, 42].

In studies of pathogen shedding in bovine milk and other animal secretions/birth products, *Brucella* was isolated in two studies. Heisch et al. (1963) isolated *B. suis* from wild rodents caught at the Kenyan coast [50]. Meundo et al. (2012) isolated five *B. melitensis* biovar 1 strains from bovine milk samples and ten *B. abortus* biovar 3 strains from aborted fetus materials and vaginal discharge fluids from cattle of the Central and Eastern provinces [41]. The cattle were from five mixed herds in which reproductive problems were reported. Figure 3a shows the summary of the number of studies by animal species in each of the Kenyan provinces and Table 2, the numbers and prevalence ranges according to livestock production system.

### Risk factors associated with human brucellosis

Our search identified six studies that measured the strength of association between potential risk factors and human brucellosis. One of them was a case control study, while the others used logistic regression models to investigate the associations of putative risk factors and *Brucella* seropositivity. Details of these studies and the corresponding odds ratios (OR) are summarized in Table 3. Generally, contact with livestock and their products were significant risk factors for *Brucella* infection. Consumption of raw milk was significantly associated with brucellosis in six out of seven counties in which studies were conducted. In these counties, the communities are mainly agro-pastoralists or predominantly nomadic pastoralists [21, 30, 32–34]. In studies conducted in Marsabit [32] and Isiolo [33], positive associations were found between seropositivity and contact with aborted materials and helping animals during birth. Further studies in Marsabit [34] and Turkana [30] revealed that contact with cattle and consumption of raw animal blood increased the risk of seropositive status. Male sex and advanced age were significant risk factors for seropositivity, while having at least basic education was protective [24, 32] (Table 3). An old case report study by Jewel, (1931) found that a patient infected with *B. abortus* had previously attended an aborting cow. The infection was subsequently linked to the cow using classical serological tests [19]. In a linked study across three livestock production systems, human brucellosis serostatus was positively correlated with the number of seropositive animals. Households that practiced the pastoral production systems and nomadic movements were at highest risk for brucellosis seropositivity. Handling of animal hides in a small holder system was a major risk factor for seropositivity [32].

**Table 3:** Summary of the studies investigating the potential risk factors showing the variables found to be associated with human brucellosis seropositivity

County	Study population characteristics	Variable	Risk factors	(aOR, 95 % CI)^a^	Ref
Marsabit	Pastoral	Individual	Age by decade Male sex Use of milk from own animals Assist in animal delivery Exposure to sheep Exposure to goats Handling of animal hides	1.1 (1.0–1.2 3.0 (2.2–4.0) 3.2 (1.7–5.8) 1.6 (1.1–2.3) 2.0 (1.4–2.8 2.1 (1.4–3.2) 1.4 (1.1–1.8)	[32]
		Household	Pastoral production system Nomadic movements Male household head Keeping sheep	42.7 (21.1–86.5) 5.7 (4.2–7.7) 2.5 (2.0–3.0) 4.0 (1.7–9.3)	
Kiambu	Small holder	Individual	Age by decade Handling of animal hides Higher education	1.6 (1.5–1.6) 83.2 (24.9–278.7) 0.1 (0.0–0.5)	
		Household	Male household head Sold livestock (1 year ago) Keeping sheep	3.0 (2.0–4.7) 2.1 (1.4–3.3) 3.5 (1.2–10.5)	
Kanjiado	Agro-pastoral	Individual	Age by decade Use of milk from own animals Regular ingestion of raw milk Exposure to sheep Handling of animal hides Higher Education	1.2 (1.2–1.4) 2.0 (1.4–3.0) 2.7 (1.9–3.9) 3.2 (2.1–5.0) 1.5 (1.2–2.0) 0.7 (0.5–0.9)	
		Household	Pastoral production system Nomadic movements Male household head Use of calving pens No exposure to aborted game	2.9 (2.1–4.0) 2.3 (1.7–3.2) 4.5 (3.4–5.9) 4.4 (1.6–11.6) 0.5 (0.2–1.2)	
National	Diverse	Individual	No education Male Advanced age (50+ years)	7.29 (1.48–35.94) 4.67 (2.37–9.19) 3.38 (1.08–10.65)	[24]
Garissa	Pastoral	Individual	Consumption of raw milk Obtaining milk from informal market	8.5 (4.20–17.26) 7.3 (2.51–21.10)	[21]
Isiolo	Pastoral	Household	Drinking raw milk Contact with aborted materials or Help during animal birth	6.57 (2.92–14.82) 1.42 (0.76–2.64) 1.27 (0.71–2.27)	[33]
Turkana	Pastoral and small holder	Individual	Pastoral production system Drinking raw blood Animal slaughter Communal grazing (exposure to goats) Communal grazing (exposure to cattle)	1.8 (*p* = 0.007) 1.4 (*p* = 0.025) 1.9 (*p* < 0.001) 1.6 (*p* = 0.003) 2.8 (*p* < 0.001)	[30]^b^
Marsabit	Pastoral	Household	Women Drinking of raw blood Consumption of raw milk Household milk harvesting	1.62 (*p* < 0.001) 1.64 (*p* < 0.001) 1.64 (*p* = 0.001) 3.87 (*p* < 0.001)	[34]^b ^

^a^adjusted Odds Ratios at 95 % Confidence Interval with *p* < 0.05

^b^95 % CI values unavailable in the study

### Brucellosis control

We found three studies that described vaccination experiments in goats, sheep, and cattle in government farms but did not qualify for data extraction during quality assessment. At the time of this review, no reports of disease incidence estimates and awareness or control programmes in Kenya were found.

## Discussion

Our work aimed to systematically review the trend of the data on brucellosis presence and frequency estimates in humans and animals, and associations between potential risk factors and human seropositivity in Kenya. The serological data reviewed in this study reveal evidence of widespread *Brucella* exposure in humans and multiple animal species in multiple regions throughout Kenya. However, despite evidence of the pervasiveness of this pathogen, we found only 36 studies/reports that were deemed of sufficient quality to provide reliable data useful to inform on disease burden estimates, or proposal of targeted disease prevention strategies based on specific risk factors. Though brucellosis was first reported in Kenya in 1916 [29], the quantity and quality of epidemiologic research for this pathogen is limited. No descriptions of disease incidence estimates appear in published literature.

In addition, our review revealed that majority of studies has limited validity that hinders the adjustment of observed apparent seroprevalences to obtain true seroprevalence. Most articles reported seroprevalence estimates from studies with unclear study designs, or sampling approaches that were likely to generate biased estimates due to non-probabilistic sampling and small sample sizes, while some studies compared the seroprevalence in purposively selected populations. Moreover, the sampling techniques used in majority of the studies did not account for the clustering of animals within the herd or flock, which may have resulted in inaccurate estimates [54]. Another critical issue with some studies is the differences in performance of the diagnostic tests used, and the lack of standardization of diagnostic tests (i.e., origin of *Brucella* antigens, control sera and the cut-off criteria). The lack of consideration of prior vaccination against brucellosis in some animal herds would also have led to misleading estimates of average prevalence.

Only one investigation reported nationwide brucellosis seroprevalence in humans and none was found for animals. Our search identified one study that used random sampling procedures in linked human and animal livestock populations [32].

To understand brucellosis epidemiology, it is essential to isolate the *Brucella* species and characterize the prevalent biovars because the available serological tests have the limitations of specificity and sensitivity.

In humans, only five studies provided bacteriological data on brucellosis. Three case-series studies described isolation of seven *B. abortus* and 66 *B. melitensis* isolates in patients treated and followed up in Nairobi and Machakos hospitals [19, 27, 29]. Except in one study, much of these data were collected more than 50 years ago and the methodologies used for pathogen cultivation and species identification are atypical or completely missing.

In animals, one study succeeded to isolate *B. suis* from wild rodents [50], but this early study reported scanty epidemiological data to allow drawing any realistic epidemiological conclusions. Recently, *B. melitensis* biovar 1 and *B. abortus* biovar 3 strains have been isolated from cattle in the Central and Eastern provinces [41]. Further genotyping revealed close molecular homology of the *B. melitensis* biovar 1 with a strain originating from Israel, and the *B. abortus* biovar 3 was closely related to a strain from neighboring Uganda suggesting a wide geographic distribution of these genotypes. Both *B. abortus* and *B. melitensis* were isolated from cattle, and none of these was found in small ruminants kept in the same herds. *B. melitensis* preferentially infects sheep and goats but may infect and persist in cattle as well [55]. Further studies are warranted to investigate the significance of cattle in brucellosis maintenance and transmission in the mixed breeding systems of Kenya.

### Brucellosis in humans

The history of brucellosis in Kenya dates back to 1916 when the first case was reported. Since then, conducted studies show high seroprevalence levels (14.4–46.5 %) in local pastoralists and agro-pastoralists (7.0–15.3 %) in the different Kenyan regions. Conversely, low levels were reported in individuals from small holder regions (0.1–2.4 %).

One recent serological study found a strong statistical association between human and animal seropositivity [32]. These findings and those from previous studies may suggest that human brucellosis is likely linked to infection of livestock species, but research gaps remain i.e., in the absence of bacteriological evidence or molecular based tests, this argument has to be interpreted with caution because serological positive results can be caused by previous exposure to infection or cross-reactivity. Additionally, co-occurrence of anti-*Brucella* antibodies in animals and humans may also suggest a common other source as opposed to source attribution. This knowledge gap highlights the need for bacteriological and molecular typing data to demonstrate pathogen reservoirs, transmission dynamics, and how *Brucella* pathogen may be embedded in livestock management practices within ecologically heterogeneous regions of Kenya.

None of the past population-based seroprevalence studies aimed to isolate the organism. A single PCR based study screened patients’ sera for *Brucella* DNA using genus specific primers [21]. Therefore, it is currently not known to what extent human brucellosis in Kenya is caused by *B. abortus* or *B. melitensis* or both*.*

Human brucellosis commonly presents as febrile illness. This makes accurate diagnosis and management in areas without access to reliable laboratory diagnosis a great challenge. The previous hospital based studies underscored the importance of laboratory supported clinical diagnosis. The studies found that patients with flu-like syndrome who were not tested for brucellosis were frequently treated for other common tropical infections [23, 26, 28, 56]. Other authors highlighted the poor performance of commonly available *Brucella* antigens for rapid agglutination test when performed without at least semi-quantitative estimation of titers or a complementary test [20, 21, 28]. In most health care facilities, brucellosis diagnosis is not routinely done and testing is considered only after the patient had initially been treated for malaria and not responded [12]. Furthermore, recent findings show that clinicians in Kenya continue to empirically treat febrile patients for malaria even after a negative rapid malaria test [57, 58]. Our search did not identify hospital based studies in Kenya’s malaria endemic zones (Nyanza and Coast provinces) [59], while one was found for the Western province [28]. It is not clear if this is likely caused by low incidence of brucellosis or if infections are unrecognized because of inaccurate diagnosis.

### Brucellosis in animals

Majority of brucellosis studies in animals were mainly serological targeting bovines and camels, and only rarely in small ruminants. Studies done before the year 2000 used RBT as the primary assay or in combination with SAT or CFT. Whereas RBT exhibits high sensitivity, the assay has poor specificity due to cross reactivity with other pathogens or failure to differentiate natural infections from the effects of vaccination [53, 60, 61]. In addition, RBT standardization and antigen origin was adequately described in 3 of 10 studies and two investigations used locally prepared antigens. Generally, ELISAs are considered to be more specific and sensitive. However, our review found that the past studies used commercial ELISA kits without prior validation of the kits under local conditions. Though sparse vaccination has been conducted in Kenyan animals before [12], only one author mentioned whether the sampled animals were vaccinated or not. Therefore, the serological data reported in these studies have to be interpreted with caution.

Kenya’s livestock population is presently estimated at 18 million beef cattle, 28 million goats, 18 million sheep, 3 million camels, 0.52 million donkeys, and 0.3 million pigs. Of these, approximately 60 % are reared in pastoralist or agro-pastoralist production and management systems [62]. Indeed, the majority of the studies were conducted in regions in which the two systems are the main economic activities. Despite livestock brucellosis being detected in animals from all livestock production systems, the seroprevalence were seemingly higher in pastoral grazing systems when compared to smallholder mixed crop or dairy farming systems. High seroprevalence i.e., 9.9–16.9 % for cattle, 11.9 % for sheep, and 13.0–16.1 % for goats were found in pastoralist managed herds [30, 32, 37, 38, 43, 45]. In contrast, seroprevalences of 0.8–2.4 % in cattle, 2.4 % in sheep, and 0–1.3 % in goats were reported in herds managed from small holder farms [32, 35, 45]. The variation may be caused by mixing of large numbers of animals, movement of livestock in search of pasture, sharing of grazing areas with wildlife, and concentration of animals around water points. Seroprevalence tended to be lower in agro-pastoralists managed herds than in pastoralist managed herds but prevalence varied (3.3–10 %) from one region to the other [26, 32, 37, 38]. This is possibly reflecting the differences in herd size and grazing patterns that highly depend on farm size [63]. Interestingly, this situation seems not to have changed over the years. For instance, three studies with intermission of 20 years done in three distinct agro-ecological zones found consistent or higher rates in extensive pastoral regions [32, 37, 45]. These findings show brucellosis in ruminant animals in most pastoral and agro-pastoral areas of the country may pose a sustained high risk for human infection.

There are only few serological studies in camels available. Additionally, these studies were largely confined to northern Kenya. The recent census report has shown that camel population in Kenya increased between 0.8 million and 3 million from 1999 to 2009 [64] causing a significant shift from subsistence to market production of camel milk for those living in the ASAL of Kenya [65]. However, this increase did not get a matched attention from public and veterinary health necessary to effectively control infectious diseases in an emerging population. Indeed, our search found only a single study on camel brucellosis since 1990. Early studies reported high seroprevalences of 10.3–38.0 % suggesting that brucellosis may be high but more extensive epidemiological research is crucial to help understand the pathogen’s ecology or prevalence in camels across Kenya.

Our search did not identify any reports of brucellosis in dogs, horses, and donkeys. Studies in several African countries, however, have reported the infection in these animals when kept in close contact with infected livestock [66–68]. This situation is also common in Kenya and the role of these animals in maintenance and transmission of brucellosis should not be underestimated. We found one small scale abattoir study reporting low prevalence (0.2 %) for pigs in central Kenya in 1976. Therefore, the situation of brucellosis in pigs still remains unknown and needs further investigation.

Wildlife surveys revealed seroprevalence of 30, 18, 14, and 5 % in African buffalo, blue wildebeest, eland, and oryx, respectively, from extensive mixed ranching-and-wildlife conservancy ranches [51, 52]. A brucellosis outbreak was also described in a commercial beef production ranch in which an abortion storm was reported in cattle and wildlife [49]. It was reported that the abortion rate increased from 5 % in 2012 to 13 % in 2013 in cattle, while six cases were detected among leopards, hyenas and lions in the same period. In addition, *B. suis* was isolated from rodents at the Kenyan coast [50]. Though the primary responsibility for diseases surveillance in domestic animals and wildlife generally rests with the respective veterinary and wildlife departments, there is need for epidemiological investigations at the wildlife/livestock interface to guide the development of effective control strategies.

### Risk factors

The reports reviewed suggest that the risk factors for human seropositivity are direct and indirect contact with livestock and their products [19, 21, 31–34, 42, 69]. Due to cultural habits and livestock husbandry practices that are common amongst pastoralist communities, the studies identified risk factors linked to direct contact with animals/products and consumption of contaminated raw milk and blood. In contrast, low risk of infection was found among individuals from intensive production systems and urban populations, and only direct contact with infected animals or contaminated products (hides) were found to be significantly associated with seropositivity. These differences can be attributed to high risk practices among pastoralists’ communities that promote brucellosis transmission such as (i) consumption of raw milk and blood, (ii) nomadic movements, (iii) using communal grazing lands and watering points for animals, and (iv) household slaughter of animals during traditional and religious ceremonies [70, 71]. Gender specific roles and responsibilities that are mainly associated with cultural practices predispose certain genders to higher risk for human brucellosis. These practices vary considerably among the multiple ethnic groups in Kenya [24, 69, 71]. However, the available information is scanty and warrant more detailed investigation.

### Control of brucellosis

Prevention and control of brucellosis in humans largely depends on successful control of the disease in livestock. These goals have been achieved in some countries by use of vaccination, test and slaughter policy and strict control of animal movement [72, 73]. The Food and Agriculture Organization (FAO) recommends different control strategies for brucellosis in livestock depending on baseline herd-seroprevalence estimates, prevailing socioeconomic conditions, surveillance and monitoring system available, and the control policy of the respective countries [74].

In Kenya, vaccination against livestock brucellosis was considered in early 1970’s and a series of vaccine trials were done in government owned herds [75, 76]. However, animal vaccination against brucellosis is rarely conducted and if done, it has been on an ad hoc basis rather than as part of a coordinated national program [12]. We found no report on human vaccination against brucellosis in Kenya.

In 2011, the Government of Kenya formed the Kenya Zoonotic Diseases Unit (ZDU). This intergovernmental unit aimed to establish collaborative programmes for effective prevention and control of zoonotic diseases in Kenya. Then, brucellosis was included as one of the priority zoonotic diseases and gazetted as a notifiable disease in Kenya under the animal diseases act the same year [77]. At the time of this review, we found no reports describing coordinated national brucellosis control programmes. Reported activities were restricted to seroprevalence studies of particular areas in Kenya. Similar to other African countries, a great challenge for control programmes is the lack of public and veterinary health services due to both decreased governmental resources and the lack of interest by the private sector to support it. For an effective control strategy in Kenya, unregulated cross-border livestock movements between neighboring countries along trade routes, and nomadic movements in search of pasture and watering points should be considered. These activities may allow the entry and spread of infected herds presenting a challenge to the internal control activities, and require collaboration at the regional level.

## Conclusion

### The way forward and key lessons for Kenya

Our review has identified major gaps in the availability of epidemiological data and diagnostic means. Brucellosis exposure is present in all animal production systems and wildlife. Human seroposivity is possibly linked to direct and indirect contact with the livestock or contaminated animal products.

It is 100 years since the disease was first reported, but little is known about the disease causing agents in Kenya. Bacteriological and molecular typing data is needed to highlight zoonotic potential, transmission dynamics, and to establish effective control measures.

Studies highlighted that patients with brucellosis are likely misdiagnosed and possibly mistreated due to lack of reliable laboratory diagnostic support and apparent ignorance of physicians. A need for increased awareness of the disease and efforts to establish reliable diagnostic capacity in the hospitals is highly recommended. Based on available evidence, it is likely that targeted vaccination of livestock populations combined with sustained surveillance and reporting systems, and public health education programmes focusing on the key socio-cultural and economic risk factors identified, may be feasible control options for the country. Eradication however seems unlikely.

The relevant authorities (veterinary health, wildlife health and public health) should initiate well-designed countrywide, evidence-based, and multidisciplinary studies of brucellosis at the human/livestock/wildlife interface to generate reliable diseases incidence estimates, potential impact, transmission dynamics, reservoirs and effective targeted control strategies.

## Abbreviations

BMAT: Brucella micro-agglutination test
CFT: Complement fixation test
ELISA: Enzyme-linked immunosorbent assay
FAO: Food and Agriculture Organization of the United Nations
HIV: Human Immunodeficiency Virus
IFA: Immuno fluorescence antibody assay
MRT: Milk ring test
q-PCR: Quantitative real-time Polymerase Chain Reaction
RBT: Rose Bengal plate test
SAT: Serum agglutination test
WHO: World Health Organization
ZDU: Zoonotic disease unit
